# Electrocardiographic tracking of left ventricular hypertrophy in hypertension: incidence and prognostic outcomes from the SPRINT trial

**DOI:** 10.1186/s40885-024-00275-8

**Published:** 2024-07-01

**Authors:** Zhuxin Zhang, Le Li, Zhenhao Zhang, Zhao Hu, Yulong Xiong, Likun Zhou, Yan Yao

**Affiliations:** Cardiac Arrhythmia Center, National Center for Cardiovascular Diseases, Fuwai Hospital, National Key Laboratory, Chinese Academy of Medical Sciences and Peking Union Medical College, Beijing, 100037 P.R. China

**Keywords:** Left ventricular hypertrophy, Hypertension, Intensive blood pressure lowering, SPRINT trial

## Abstract

**Background:**

This study explores the impact of intensive blood pressure (BP) control on left ventricular hypertrophy (LVH) incidence and evaluates the prognostic implications of LVH status (pre-existing/new-onset/persistent/regression) using Systolic Blood Pressure Intervention Trial (SPRINT) Electrocardiogram Data.

**Methods:**

Poisson regression was used to assess new-onset LVH and LVH regression rates. Multivariable-adjusted Cox proportional hazard models determined the risk of adverse cardiovascular events (ACE), a composite of myocardial infarction (MI), non-MI acute coronary syndrome, stroke, heart failure, or cardiovascular death, alongside safety adverse events.

**Results:**

In 8,016 participants, intensive BP control significantly reduced new-onset LVH (8.27 vs. 14.79 per 1000-person years; adjusted *p*<0.001) and increased LVH regression (14.89 vs. 11.89 per 1000-person years; adjusted *p*<0.001). Elevated ACE risk was notable in participants with pre-existing LVH [adjusted HR: 1.94 (95% CI: 1.25–2.99); *p* = 0.003], new-onset LVH [adjusted 1.74 (95% CI: 1.16–2.60); *p* = 0.007], and persistent LVH[adjusted HR: 1.96 (95% CI: 1.11–3.46); *p* = 0.020], compared to those without LVH. Intriguingly, LVH regression attenuated this risk increment [adjusted HR: 1.57 (95% CI: 0.98–2.53); *p* = 0.062]. Achieving a BP target of < 120/80 mmHg nullified the increased ACE risk in those with pre-existing LVH.

**Conclusions:**

Intensive BP control is instrumental in both reducing the emergence of LVH and fostering its regression. Pre-existing, new-onset LVH and persistent LV remain a predictor of adverse cardiovascular prognosis, whereas LVH regression and achieving on-treatment BP < 120/80 mmHg in pre-existing LVH individuals may further mitigate residual cardiovascular risk.

**Clinical trial registration:**

URL: ClinicalTrials.gov Unique Identifier: NCT01206062.

**Supplementary Information:**

The online version contains supplementary material available at 10.1186/s40885-024-00275-8.

## Graphical abstract

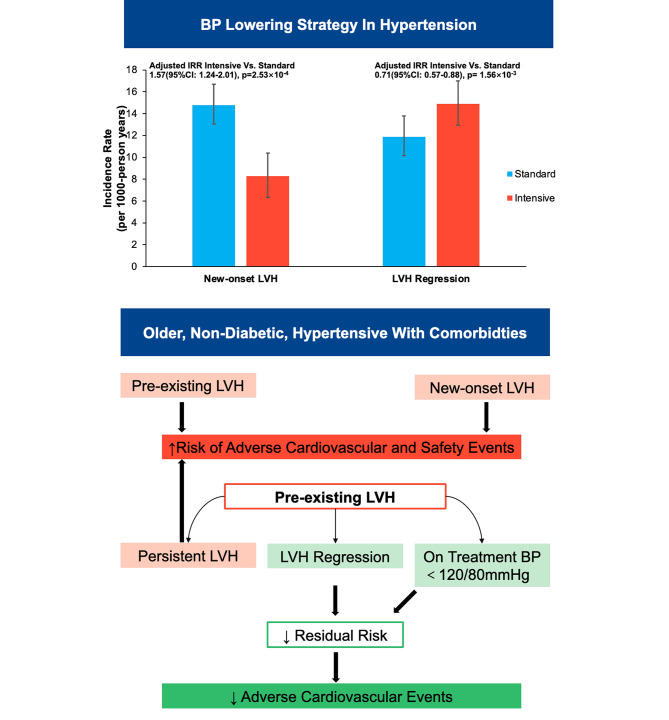

## Introduction

The left ventricle (LV) stands as a principal target for hypertensive (HTN) end-organ damage, with approximately 40% of hypertensive individuals manifesting left ventricular hypertrophy (LVH) [1]. LVH arises from the interplay of blood pressure (BP)-induced morphological transformations and subclinical pathophysiological shifts within the myocardium [1]. Variations in left ventricular geometry—concentric remodeling, and concentric or eccentric hypertrophy—are now recognized as potent independent predictors of cardiovascular outcomes, beyond their traditional role as markers of hypertensive severity [2, 3]. The ACC/AHA High Blood Pressure guideline recognizes the dual prognostic effects of LV geometric changes but refrains from endorsing routine echocardiography or cardiac MRI for LVH assessment in hypertension management, citing concerns about cost-effectiveness [4]. Despite the diminished sensitivity and specificity of electrocardiographic LVH diagnosis, it remains firmly established for prognostic purposes in cardiovascular disease [5, 6]. 

While the role of elevated BP in LVH pathogenesis is acknowledged [7, 8], the impact of blood pressure modulation on LVH dynamics remains underexplored in randomized settings [9]. The observational studies also offered inconsistent insights into the benefits of standard (SBP < 140 mmHg) versus intensive (SBP < 120 mmHg) BP control in mitigating LVH [10–12]. Moreover, the influence of treatment-induced LV geometric changes on cardiovascular outcomes is still under debate, given the divergent findings reported [13, 14]. 

The Systolic Blood Pressure Intervention Trial (SPRINT) [15], a pivotal study demonstrating the advantages of intensive BP management in reducing adverse cardiovascular outcomes, provides a prime opportunity to dissect the effects of intensive BP control on LVH incidence and regression. In this study, our hypotheses are as follows: (a) Intensive BP control diminishes the risk of new-onset LVH and enhances LVH regression compared to standard BP control; (b) The presence of both new-onset and pre-existing LVH in hypertensive individuals signifies adverse prognostic implications; (c) Change in LVH status (i.e., LVH regression) contributes to reducing residual cardiovascular risks; and (d) Maintaining systolic blood pressure (SBP) < 120/80 mmHg in individuals with pre-existing LVH attenuates adverse cardiovascular events.

## Methods

### Study population and design

SPRINT constituted a randomized, controlled, open-label trial conducted across 102 clinical sites organized into five clinical center networks in the United States. The detailed rationale and design of the SPRINT trial have been previously published [15, 16]. In essence, SPRINT aimed to assess whether reducing systolic blood pressure (SBP) to < 120 mmHg would result in a reduction of cardiovascular disease (CVD) events, defined as a composite outcome encompassing non-fatal myocardial infarction (MI), non-MI acute coronary syndrome, non-fatal stroke, non-fatal acute decompensated heart failure, and death from CVD (SPRINT primary outcome). Participants needed to meet specific criteria, including an age of at least 50 years, SBP between 130 and 180 mmHg, and an increased risk of CVD (determined by the presence of clinical or subclinical CVD, chronic kidney disease, a 10-year risk of CVD ≥ 15% estimated by the Framingham risk score, or an age ≥ 75 years). Patients with type 2 diabetes mellitus or a history of stroke were excluded from the study. A total of 9,361 participants were enrolled between November 2010 and March 2013, with 4,683 randomized to SBP target of < 140 mmHg (standard treatment arm) and 4,678 participants randomized to < 120 mm Hg (intensive treatment arm). Randomization was stratified by clinical site. The SPRINT intervention was terminated early (median 3.26 years of follow-up) due to a 25% reduction in the primary composite CV endpoint and a 27% reduction in all-cause mortality in the intensive treatment group. The study received approval from the institutional review board at each participating site, and written informed consent was obtained from all participants.

For this analysis, we included SPRINT participants with baseline electrocardiogram (ECG) data obtained within the first 7 days and at least one follow-up ECG. The 7-day timeframe is to ensure the collected ECG data accurately represent each participant’s cardiovascular status at the onset of the study. Participants with missing or uninterpretable baseline ECG (*n* = 307) and those without any follow-up ECG (*n* = 1,038) were excluded from the analysis. In this study, participants were classified based on LVH status at initial and subsequent ECG screenings into the following groups: Pre-existing LVH refers to those with LVH at the first ECG; New-onset LVH applies to those who developed LVH during the study; Free of LVH includes those who had no LVH initially and remained free of hypertrophy throughout the study period; Persistent LVH captures those with LVH at baseline that persisted across all follow-ups; LVH Regression denotes individuals presenting with LVH initially but showing reduction on later ECGs; and No LVH describes participants without LVH at baseline. The data supporting the findings of this study are accessible from the National Heart, Lung, and Blood Institute Biologic Specimen and Data Repositories upon application.

### Electrocardiographic LVH ascertainment

LVH determination was based on standard 12-lead ECG obtained at baseline, year 2, year 4, and the close-out visit. Digital ECG data were recorded using a GE MAC 1200 electrocardiograph (GE, Milwaukee, Wisconsin) at 10 mm/mV calibration and a speed of 25 mm/s. Centralized ECG reading was conducted at the Epidemiological Cardiology Research Center (EPICARE), Wake Forest School of Medicine, Winston-Salem, North Carolina. Initial visual inspection of all ECG tracings ensured the absence of technical errors and maintained adequate quality before automated processing using GE 12-SL Marquette version 2001 (GE, Milwaukee, Wisconsin). LVH was defined using the Cornell voltage criteria (RaVL amplitude + SV3 amplitude) with sex-specific cut-off points: ≥2,200 microvolt (µV) in women and ≥ 2800 µV in men [17]. The categorization of LVH as present or absent (or changed status) was determined by crossing these cut-off points either up or down (indicating regression or progression) even by a single point. Additionally, Cornell voltage was analyzed as a continuous variable, providing the advantage of not relying on specific cut-off points to define LVH and being more sensitive to changes during follow-up compared to LVH as a categorical variable.

In sensitivity analysis, we applied the Cornell voltage product ([RaVL amplitude + SV3 amplitude] × QRS duration) LVH criteria [18]. Another approach involved using LVH by Minnesota Code ECG classification, which incorporates LVH criteria with ST/T abnormalities (LVH with a strain pattern) in selected analyses that do not necessitate the use of continuous measures like Cornell Voltage. Minnesota Code LVH is defined as high-amplitude R waves (Minnesota Code 3.1: R amplitude > 26 mm in either V5 or V6, or R amplitude > 20.0 mm in any of leads I, II, m, aVF, or R amplitude > 12.0 mm in lead aVL) plus major ST/T abnormalities (Minnesota Codes 4.1, 4.2, 5.1, or 5.2) [19]. 

### Outcomes

Our primary objective was to assess the risk of adverse cardiovascular events (ACE), a composite of myocardial infarction (MI), non-MI acute coronary syndrome, stroke, heart failure, or cardiovascular cause of death. Secondary outcomes encompassed an examination of individual components of the primary outcome, all-cause mortality, and a composite outcome combining the primary events with all-cause mortality. Furthermore, safety adverse events (SAE) were evaluated as a secondary clinical outcome, which included serious adverse events and specific adverse events of hypotension, syncope, bradycardia, electrolyte imbalance, injurious fall, acute kidney injury, low sodium, low potassium, and excessive potassium.

In terms of secondary subclinical outcomes, our analysis included an assessment of the incidence and regression of LVH throughout the total trial follow-up period. New-onset LVH was defined when individuals without baseline LVH met Cornell voltage criteria for LVH on a follow-up ECG. Conversely, LVH regression occurred when individuals with baseline LVH no longer met Cornell voltage criteria for LVH on a follow-up ECG. Follow-up time was censored on the date of the last ECG. Additionally, we explored the annual change in Cornell voltage as a continuous variable, representing the annualized difference between the Cornell voltage on the last available ECG and the baseline ECG.

All cardiovascular outcomes underwent independent adjudication, and the adjudication process has been comprehensively described in previous publications [15, 16]. Only the first event for each outcome was included in these analyses.

### Statistical analyses

To delineate the impact of LVH status on cardiovascular outcomes, we stratified participants into no LVH, new-onset LVH, and pre-existing LVH groups. Continuous variables were presented as median and interquartile range (IQR) and compared using the Wilcoxon rank-sum test. Categorical variables were summarized as counts and percentages, with comparisons made using the χ^2^ test. Incidence rates of new-onset LVH were derived using Poisson regression, with multivariable adjustments to account for a broad range of covariates, including age, sex, race, body mass index, baseline systolic and diastolic blood pressure, baseline Cornell voltage, clinical or subclinical cardiovascular disease, heart failure, atrial fibrillation, kidney function, plasma metabolic markers, medication use, smoking status, alcohol abuse, and treatment strategy. These analyses informed the generation of adjusted Kaplan-Meier curves that illustrate the cumulative incidence stratified by treatment approach. The relative strength of association between the risk of new-onset LVH/ LVH regression and various clinical and demographic factors was evaluated using multivariable-adjusted Cox models, as described previously [20]. Similarly, we used Cox models to evaluate the risk of ACE and secondary outcomes among different groups categorized by LVH status, modifying for the same covariates and introducing multiplicative interaction terms to test treatment strategy influences.

Several additional analyses were conducted. We compared the rate of regression of mean Cornell Voltage during follow-up between the intensive and standard arms using linear models, with random slope and intercept models to account for individual variations over time. We also corroborated these findings using alternative LVH criteria, namely the Cornell voltage product and the Minnesota Code, for consistency in LVH classification. All statistical analyses were performed using R version 4.0.2. Proportional hazard assumptions for Cox regression models were checked and met. Reported *p*-values are two-sided, and statistical significance was set at a threshold of 5%.

## Results

### Incidence of new-onset LVH and LVH regression in 2 treatment arms

**Fig. 1:**
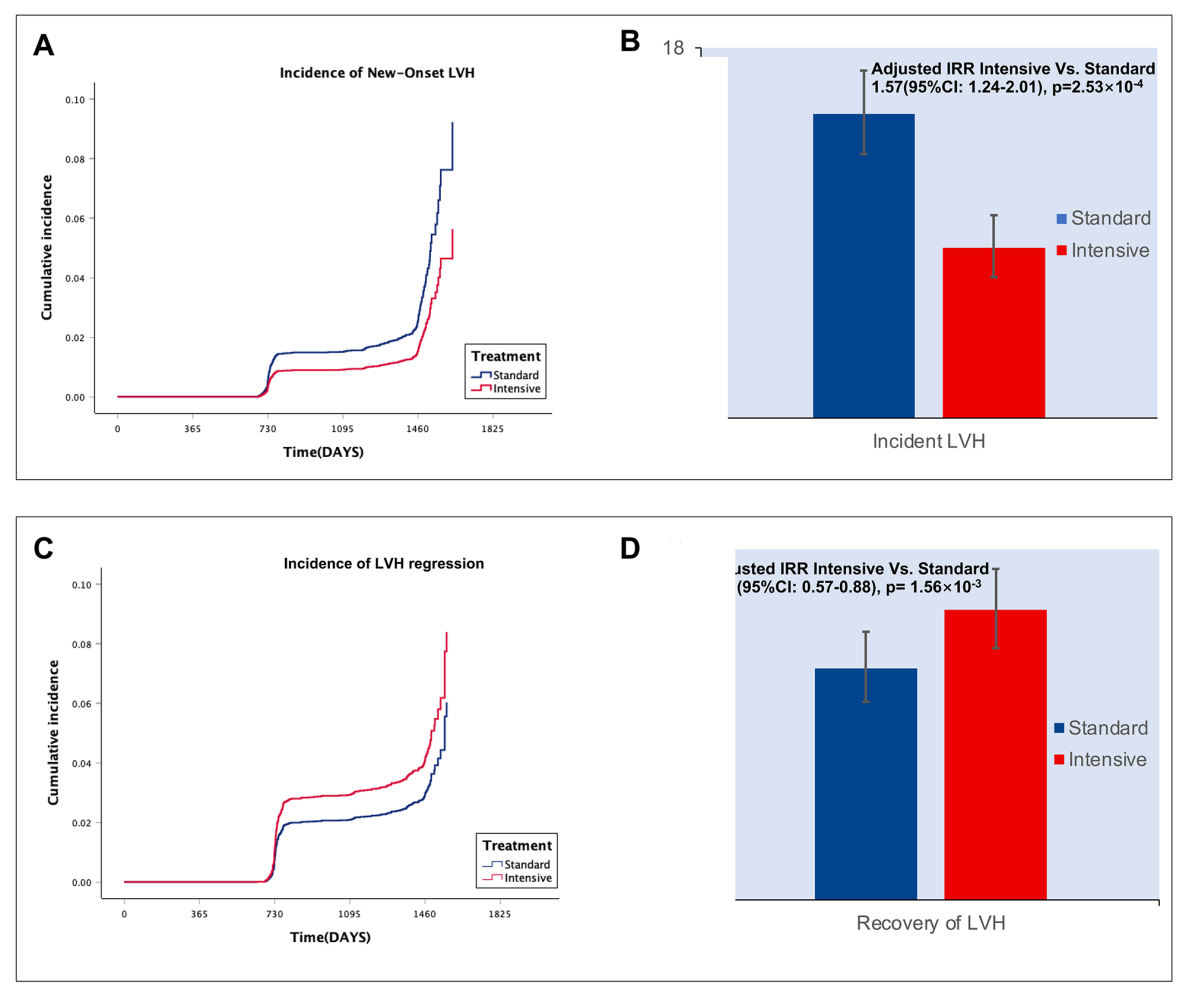
Incidence of new-onset LVH and LVH regression: stratified by treatment strategy. Panel **A** and **C**: Adjusted cumulative incidence of new-onset LVH and LVH regression by treatment strategy. Panel **B** and **D**: Incidence rate of LVH in participants randomized to intensive (red) and standard (blue) blood pressure management. LVH: left ventricular hypertrophy; IRR: incidence rate ratio

In the cohort of 8,016 participants with available data, 7.45% had pre-existing LVH. The follow-up duration for ECG assessments ranged from 7 to 1903 days, with an average follow-up time of 1293.74 days (3.54 years). The incidence rates for new-onset LVH and LVH regression were 11.52 (95% CI: 10.30–12.83) and 13.39 (95% CI: 12.08–14.80) per 1000-person years, respectively. The average duration of LVH progression among new-onset LVH patients was approximately 998.91 days. Participants who developed new-onset LVH were characterized by advanced age [71 (IQR: 62–78) years vs. 67 (IQR: 61–75) years; *p* < 0.001], a higher proportion of black individuals (46.6% vs. 28.8%; *p* < 0.001), and a higher prevalence of chronic kidney disease (36.7% vs. 27.0%; *p* < 0.001) and atrial fibrillation (14.2% vs. 7.4%; *p* < 0.001) compared to those without LVH during the study period. Patients with pre-existing LVH, in comparison to those without LVH, were more likely to be black (51.8% vs. 28.8%; *p* < 0.001) and female (51.9% vs. 32.4%; *p* < 0.001). Additionally, patients with pre-existing LVH had a higher prevalence of clinical and subclinical cardiovascular disease (27.1% vs. 18.9%, *p* < 0.001). Heart failure was more prevalent in both pre-existing and new-onset LVH groups. Baseline SBP and diastolic blood pressure (DBP) levels were significantly higher in individuals with pre-existing LVH compared with those without LVH (Table 1, Supplementary Materials Table S1). Distinct rates of new-onset LVH and LVH regression were observed in the two treatment arms. The incidence rates for new-onset LVH were 14.79 (95% CI: 12.85–16.91) per 1000-person years in the standard arm and 8.27 (95% CI: 6.85–9.88) per 1000-person years in the intensive arm. Conversely, the rate of recovery from LVH was higher in the intensive arm compared to the standard arm, with rates of 14.89 (95% CI: 12.95–17.01) and 11.89 (95% CI: 10.16–13.80) per 1000-person years, respectively. The multivariable-adjusted incidence rate ratio for new-onset LVH revealed a 1.57-fold higher risk in the standard arm compared to the intensive arm (95% CI: 1.24–2.01; *p* = 2.53 × 10^− 4^). In contrast, for LVH regression, the incidence rate ratio of the standard arm vs. the intensive arm was 0.71 (95% CI: 0.57–0.88; *p* = 1.56 × 10^− 3^), indicating a higher probability of LVH regression in the intensive treatment group (Fig. 1, Supplementary Materials Table S2).

**Table 1:** Baseline characteristics

Characteristics*	Free of LVH (*n* = 7100)	New-onset LVH (*n* = 319)	Pre-existing LVH (*n* = 597)	*p*
Age, y [IQR]	67.00 [61.00, 75.00]	71.00 [62.00, 78.00]	68.00 [60.00, 75.00]	< 0.001
Age ≥ 75 y, n (%)	1924 (27.1)	125 (39.2)	168 (28.1)	< 0.001
Female, n (%)	2302 (32.4)	175 (54.9)	353 (59.1)	< 0.001
Black, n (%)	2044 (28.8)	148 (46.4)	309 (51.8)	< 0.001
Current Smoking, n (%)	959 (13.5)	40 (12.6)	82 (13.8)	0.872
Alcohol Abuse, n (%)	284 (4.0)	8 (2.5)	14 (2.3)	0.059
Body mass index, kg/m2 [IQR]	29.04 [25.96, 32.88]	28.57 [25.74, 33.40]	29.06 [25.53, 32.95]	0.741
Systolic blood pressure, mm Hg [IQR]	138.00 [129.00, 148.00]	138.00 [129.00, 150.00]	144.00 [134.00, 158.00]	< 0.001
Diastolic blood pressure, mm Hg [IQR]	78.00 [70.00, 86.00]	76.00 [68.00, 84.00]	80.00 [71.00, 88.00]	< 0.001
Blood pressure medications, n	2.00 [1.00, 2.00]	2.00 [1.00, 3.00]	2.00 [1.00, 3.00]	< 0.001
Serum creatinine, mg/dL [IQR]	1.01 [0.86, 1.20]	0.99 [0.82, 1.22]	0.99 [0.83, 1.23]	0.213
Estimated GFR, mL/min/ 1.73m2 [IQR]	71.53 [58.76, 84.74]	70.00 [54.62, 84.59]	70.29 [56.82, 84.03]	0.065
Urine albumin/creatinine, mg/g [IQR]	9.18 [5.56, 20.00]	11.04 [6.07, 24.88]	13.01 [6.94, 32.66]	< 0.001
Chronic kidney disease†, n (%)	1918 (27.0)	117 (36.7)	181 (30.3)	< 0.001
Total cholesterol, mg/dL [IQR]	186.00 [161.00, 214.00]	191.00 [162.00, 216.00]	191.00 [166.00, 222.00]	0.004
High-density lipoprotein cholesterol, mg/dL [IQR]	50.00 [43.00, 60.00]	52.00 [44.00, 64.00]	53.00 [44.00, 64.00]	< 0.001
Triglycerides, mg/dL [IQR]	108.00 [78.00, 152.00]	100.00 [74.50, 140.50]	99.00 [74.00, 138.00]	< 0.001
Fasting plasma glucose, mg/dL [IQR]	98.00 [91.00, 105.00]	96.00 [88.00, 103.00]	96.00 [90.00, 104.00]	< 0.001
Clinical/subclinical cardiovascular disease, n (%)	1345 (18.9)	78 (24.5)	162 (27.1)	< 0.001
Heart Failure, n (%)	204 (2.9)	28 (8.8)	33 (5.5)	< 0.001
Atrial Fibrillation, n (%)	528 (7.4)	45 (14.2)	54 (9.1)	< 0.001
Statin Use, n (%)	3148 (44.6)	130 (41.0)	228 (38.5)	0.009
Aspirin Use, n (%)	3656 (51.6)	157 (49.5)	289 (48.5)	0.284
Cornell voltage,µV [IQR]	1407.00 [1051.00, 1765.25]	1975.00 [1610.50, 2174.50]	2858.00 [2452.00, 3178.00]	< 0.001

*Categorical variables are represented as counts with proportions and continuous variables are represented as medians with interquartile ranges [IQR].

†Defined as baseline estimated glomerular filtration rate < 60 ml/min/1.73m^2^

Linear regression models were employed to assess the treatment effect on annual changes in Cornell voltage, stratified by LVH category and treatment group. The rate of regression of the Cornell voltage (the sum of the amplitude of RaVL + SV3) was faster in the intensive BP lowering arm than in the standard arm by -36.30 µV/year (95% CI: -43.13 to -29.48) (*p* = 2.53 × 10^− 25^) in the overall population. Intensive SBP lowering led to a more pronounced reduction in the Cornell voltage during the follow-up period across both LVH categories (Table 2). The effect of intensive SBP lowering on the reduction in Cornell voltage index was significantly stronger among pre-existing LVH participants compared with free of LVH participants (P for interaction = 4.34 × 10^− 4^) (Table 2). Throughout the trial course, a sustained between-group difference in Cornell voltage persisted in patients with and without LVH (Supplementary Materials Figure S1). At 1 year, the mean between-group difference in Cornell voltage was 234.0 and 57.4 µV in patients with and without LVH, respectively. After the trial was stopped, BP management decisions were returned to the primary care physicians, resulting in a reduction of the CV difference between the treatment groups.

**Table 2:** Effect of intensive SBP lowering on mean change in Cornell voltage stratified by combined LVH status at baseline

LVH status	Standard SBP Group mean change in CV per year (*n* = 4004)	Intensive SBP Group mean change in CV per year (*n* = 4012)	Difference in mean change in CV per year		*P* for interaction
	(95% CI)	(95% CI)	(95% CI)	*p*	
Overall	-4.22 (-9.11, 0.66)	-40.53 (-45.29, -35.76)	-36.30 (-43.13, -29.48)	2.53 × 10^− 25^	*-*
LVH -	3.29 (-1.48, 8.07)	-29.66 (-34.12, -25.21)	-32.96 (-39.49, -26.42)	6.25 × 10^− 23^	0.000436
LVH +	-97.26 (-123.05, -71.46)	-175.79 (-203.66, -147.91)	-78.53 (-116.43, -40.63)	5.36 × 10^− 5^	

Abbreviations: CI: confidence interval; CV: Cornell voltage; LVH: left ventricular hypertrophy; SBP: systolic blood pressure

In sensitivity analyses using Cornell voltage product and Minnesota Code LVH criteria, the effects of intensive SBP lowering on clinical and LVH outcomes were overall similar to using Cornell voltage index in the main analyses (Supplemental Materials Tables S2).

**Ranking of the relative strength of association of risk factors with new-onset LVH and LVH regression**.

**Fig. 2:**
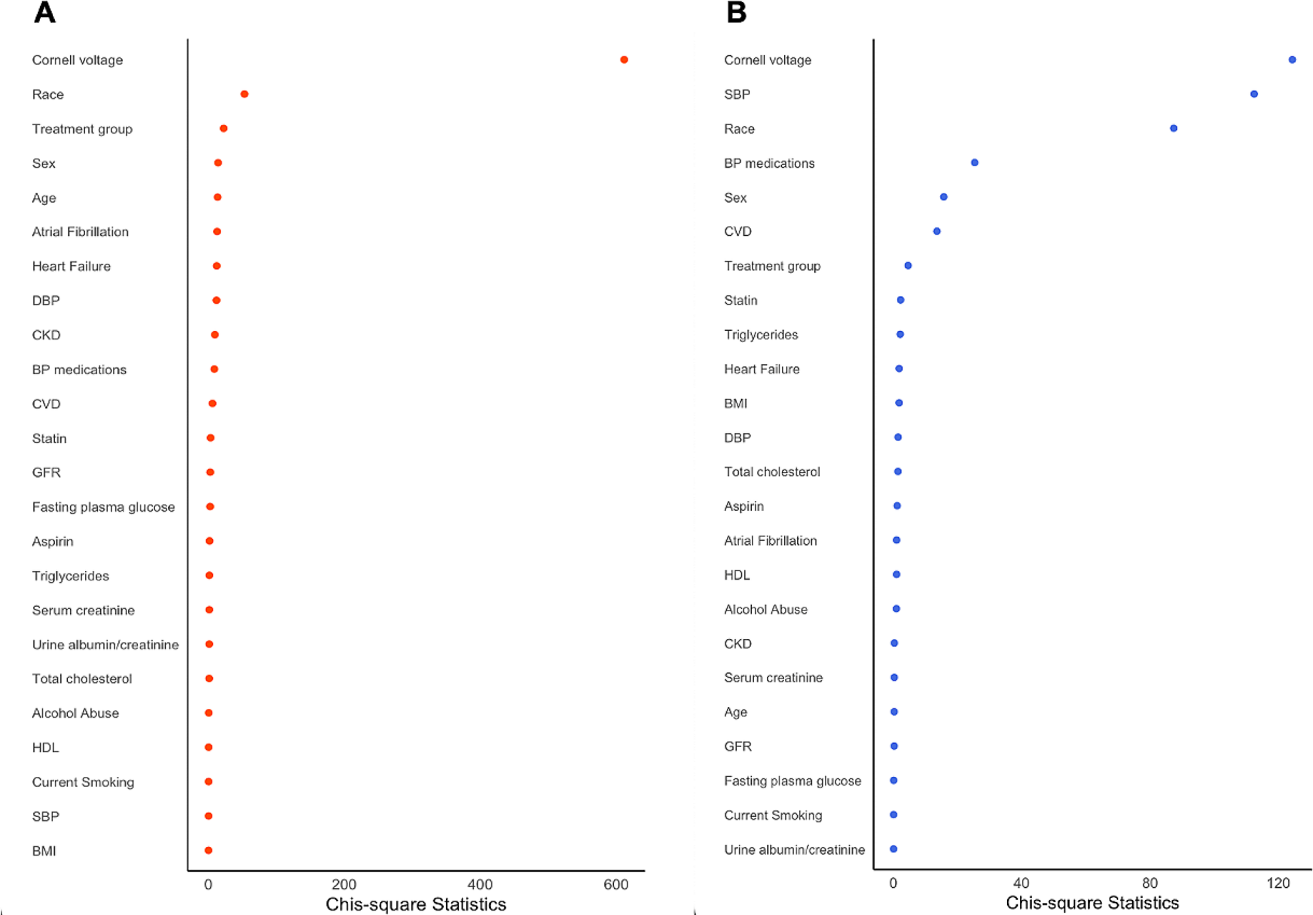
Ranking of strength of association between new-onset LVH/ LVH regression and clinical and demographic factors. Panel **A**: New-onset LVH. Panel **B**: LVH regression. These figures demonstrate the relative strength of associations of respective covariates, ranked according to chi-squared values from the multivariate-adjusted model. Chi-square values were corrected for degrees of freedom allocated to respective covariates in the model ensuring comparison on the same scale

Figure 2 outlines the importance of various risk factors for new-onset LVH and regression using the partial chi-square statistic (χ2). For new-onset LVH, the most significant predictors are Cornell voltage (χ2 = 612.52), followed by demographic and clinical factors such as race, treatment group, and cardiovascular health markers. For LVH regression, the leading factor remains Cornell voltage (χ2 = 124.34), with systolic blood pressure and other clinical conditions also showing substantial associations.

### Prognostic implications of new-onset and pre-existing LVH

**Fig. 3:**
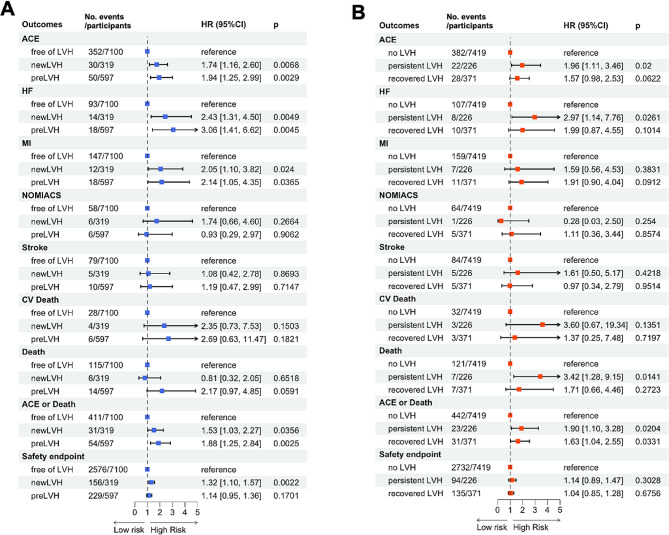
Implications of LVH status on cardiovascular and safety outcomes. Panel **A**: Prognostic implications of new-onset and pre-existing LVH. Panel **B**: Prognostic implications of persistent LVH and LVH regression. newLVH refers to new-onset LVH; preLVH refers to pre-existing LVH; recovered LVH refers to LVH regression. Definitions of each LVH group can be found in Method section. HR, hazard ratio; CI, confidence interval; LVH, left ventricular hypertrophy; ACE, adverse cardiovascular events; HF, heart failure; MI, myocardial infarction; NOMIACS, non-MI acute coronary syndrome; CV, cardiovascular

ACE occurred in 5.0% of participants free of LVH. In comparison, incidences were higher at 8.4% for those with pre-existing LVH and 9.4% for individuals with new-onset LVH. (Fig. 3A). The secondary endpoint revealed the proportion of participants in each group who experienced stroke, myocardial infarction (MI), non-MI acute coronary syndrome, heart failure, cardiovascular death, all-cause death, and safety adverse events (Fig. 3A). Participants with pre-existing LVH [1.94 (95% CI: 1.25–2.99); *p* = 0.003] and new-onset LVH [HR: 1.74 (95% CI: 1.16–2.60); *p* = 0.007] had a higher hazard for the development of ACE compared to those free of LVH (Fig. 3A). No interaction by treatment strategy was observed (*p* > 0.5). In individuals with new-onset LVH receiving standard treatment, the hazard for the development of ACE was 1.21 [(95% CI: 0.52–2.81); *p* = 0.664] compared to intensive therapy, as detailed in Fig. 3A. The hazard for HF in those with pre-existing LVH was 3.06 (95% CI: 1.41–6.62; *p* = 0.005), and for those with new-onset LVH, it was 2.43 [(95% CI: 1.31–4.50); *p* = 0.005]. Individuals with pre-existing LVH [HR: 2.14 (95% CI: 1.05–4.35); *p* = 0.037] and new-onset LVH [HR: 2.05 (95% CI: 1.10–3.82); *p* = 0.024] also had a greater risk of developing MI compared to those free of LVH. The hazard for ACE or all-cause death was 1.88 (95% CI: 1.25–2.84; *p* = 0.003) for pre-existing LVH and 1.53 (95% CI: 1.03–2.27; *p* = 0.036) for those with new-onset LVH. Notably, the risk of SAE was higher in new-onset LVH [1.32 (95% CI: 1.10–1.57); *p* = 0.002], but not in pre-existing LVH [1.14 (95% CI: 0.65–1.36); *p* = 0.170] (Fig. 3A). Intensive BP treatment did not increase the risk of SAE in patients with pre-existing LVH (HR, 1.07; 95% CI, 0.81–1.41), new-onset LVH (HR, 0.92; 95% CI, 0.63–1.33), and free of LVH (HR, 1.04; 95% CI, 0.96–1.12). We also observed similar results in sensitivity analyses using other commonly used LVH criteria (Cornell voltage product and Minnesota Code) (Supplemental Materials Tables S3).

### Prognostic significance of LVH status change during treatment of hypertension

Participants with persistent LVH [1.96 (95% CI: 1.11–3.46); *p* = 0.020] had a higher hazard for the development of ACE compared to no LVH at baseline (Fig. 3B). However, recovery from LVH [HR: 1.57 (95% CI: 0.98–2.53); *p* = 0.062] was no longer associated with an increased hazard for ACE. No interaction by treatment strategy was observed (*p* > 0.1). In individuals with persistent LVH on standard treatment, the hazard for the development of ACE was 1.06 [(95% CI: 0.38–2.94); *p* = 0.910] compared to intensive therapy. The hazard for HF in those with persistent LVH was 2.97 (95% CI: 1.14–7.76; *p* = 0.026). For LVH regression, the association attenuated to null [HR: 1.99 (95% CI: 0.87–4.55); *p* = 0.101]. The hazard for ACE or all-cause death was 1.90 (95% CI: 1.10–3.28; *p* = 0.020) for persistent LVH and 1.63 (95% CI: 1.04–2.55; *p* = 0.033) for LVH regression. Notably, the risk of all-cause death was higher in those with persistent LVH [3.42 (95% CI: 1.28–9.15); *p* = 0.014] but not in those with LVH regression [1.71 (95% CI: 0.66–4.46); *p* = 0.272] (Fig. 3B). Sensitivity analyses using two other LVH criteria showed similar results (Supplemental Materials Tables S4).

### Blood pressure control and residual cardiovascular risk in patients with pre-existing LVH

**Fig. 4:**
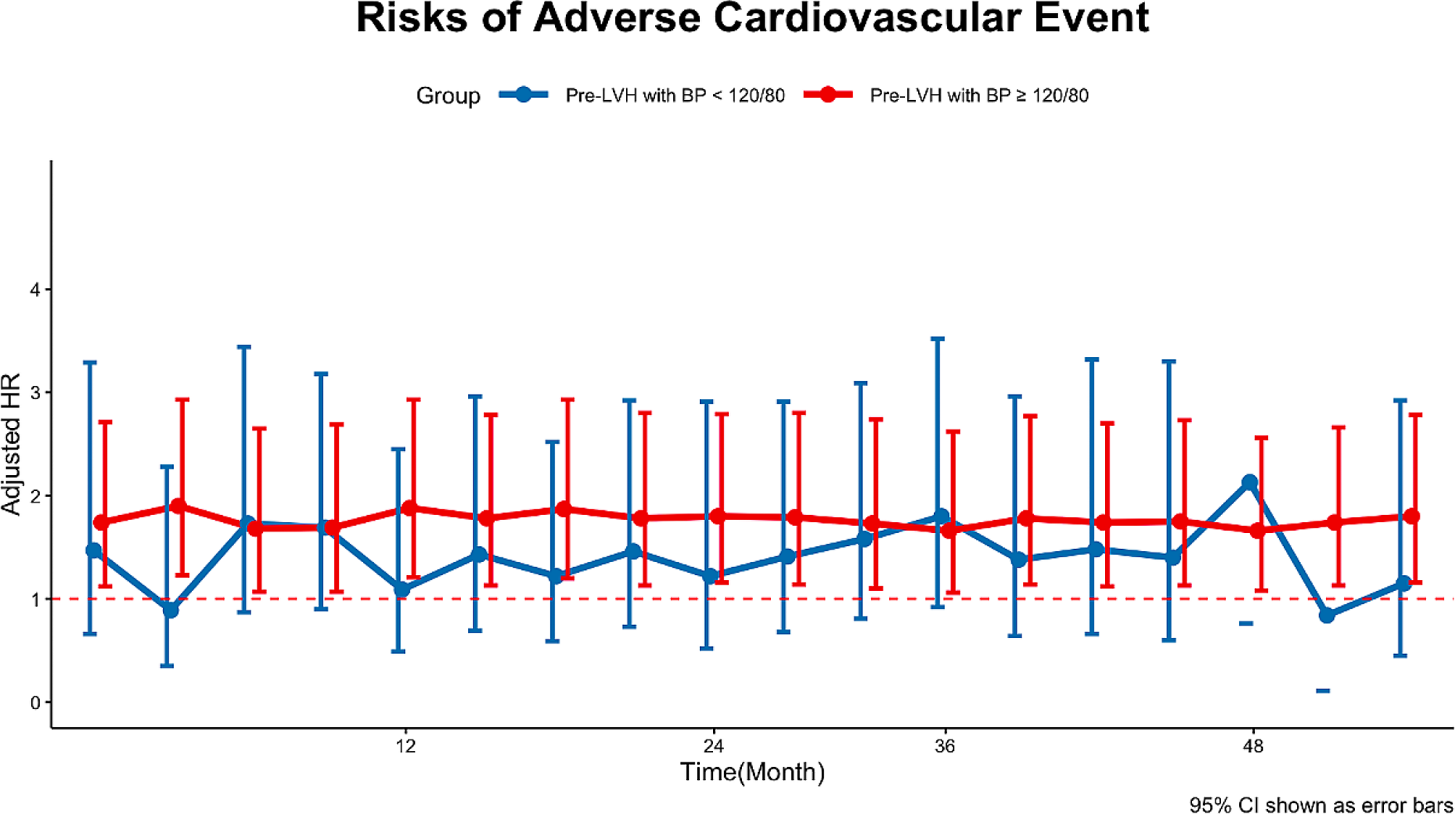
Residual cardiovascular risk in participants with pre-existing LVH for the risk of development of adverse cardiovascular events. Those with pre-existing LVH and having attained blood pressure < 120/80 mmHg at each visit are represented in blue and those who did not are represented in red. I bars represent 95% confidence intervals. HR, hazard ratio; LVH, left ventricular hypertrophy; Pre-LVH, pre-existing LVH

Participants with LVH at baseline were categorized into a normal BP group and an abnormal BP group based on whether the BP values were within the normal range (< 120/80 mm Hg) at each follow-up visit. We investigated whether there were differences in the primary endpoints between these groups. Notably, participants with pre-existing LVH and consistently high BP (≥ 120/80 mmHg at each checkpoint) faced greater risks of ACE than those without LVH. Conversely, those achieving BP control showed similar ACE risks to participants without LVH (Fig. 4).

## Discussion

Our findings reveal that intensive BP management curtails the onset of LVH and fosters the regression of established LVH, underscoring LVH’s prognostic importance. LVH, irrespective of its genesis or persistence, is linked with increased cardiovascular risk, but optimal BP control (< 120/80 mmHg) can mitigate this in those with pre-existing LVH. These findings emphasize the crucial role of intensive BP control in preventing LVH development and improving cardiovascular outcomes.

Previous investigations have elucidated the potential for BP reduction to induce regression in LVH [21, 22], though not uniformly [23], and with the possibility of new-onset LVH despite optimal BP levels [24–26]. Our analysis within the Systolic Blood Pressure Intervention Trial (SPRINT) extends these findings, demonstrating the incremental benefit of intensive BP management in a high-risk, non-diabetic hypertensive cohort. Participants under standard BP control (SBP < 140 mmHg) experienced higher rates of new-onset LVH, while intensive control (SBP < 120 mmHg) facilitated notable LVH regression, particularly in those with pre-existing conditions. The impact of pre-existing cardiovascular co-morbidities, such as atrial fibrillation and heart failure, coupled with the presence of clinical or subclinical cardiovascular disease, surfaced as robust indicators for the onset of new LVH. The administration of antihypertensive medications and the achieved SBP levels were critical in promoting recovery from LVH, supporting the hypothesis that BP control plays a significant role in mitigating pathological cardiac remodeling. Effective and prolonged BP control might interfere with or inhibit structural remodeling, autonomic dysregulation, increased oxidative stress, interstitial fibrosis, and activation of the renin-angiotensin-aldosterone system which have been proposed as probable pathophysiological pathways for LVH development in hypertension [7, 8, 27, 28]. The time needed for these therapeutic effects to manifest and the continued subclinical damage from other pathological mechanisms, however, might contribute to the residual cardiovascular risk, potentially accounting for the variability in study outcomes. The Cornell voltage, a key metric in our study, was pivotal in both new-onset and regression of LVH, with the most substantial reductions observed in patients with baseline LVH. This underscores the potential of antihypertensive treatment not just to halt but to partially reverse adverse cardiac changes by reducing afterload.

LVH is well-established as being linked to a higher incidence of cardiovascular events and mortality, whether detected by ECG or imaging [11, 29, 30], Yet, the prognostic significance of LVH—and its alteration independently from BP reduction—is contested. While some cohort studies suggest LVH regression aligns with decreased cardiovascular events [13, 22, 31], others argue LVH regression may merely reflect effective BP management rather than serving as a direct prognostic marker [14, 23, 32]. Our research highlights that LVH, alongside hypertension, elevates the risk of ACE regardless of the BP control achieved. We find that both established and newly developed LVH amplify ACE risk. Nonetheless, optimal BP control in participants with pre-existing LVH may contribute to reducing residual cardiovascular risk. Consistently high BP (≥ 120/80 mmHg) correlates with increased ACE risk. In our results, the heightened risk of SAE in new-onset LVH suggests that close monitoring and potentially more aggressive or tailored treatment strategies may be warranted for these patients. Conversely, the lack of increased risk in pre-existing LVH may suggest a physiological accommodation to the hypertrophic state or a ceiling effect of therapeutic intervention. The lack of associated risk increase from intensive BP management affirms its safety and bolsters its role in mitigating cardiovascular event risk, thereby informing the nuanced risk-benefit profile of intensive hypertension therapy.

Our study further establishes that alterations in LVH status serve as significant prognostic factors. Among baseline LVH patients, two-thirds showed regression with antihypertensive treatment. Notably, recovery from LVH, while not associated with improved outcomes, might eliminate the risk of ACE compared to persistent LVH. Yet, some patients, despite adequate BP control, experienced irreversible LV remodeling, indicative of a grimmer prognosis. The persistence of LVH is an independent predictor for ACE and mortality, underscoring the need for vigilant monitoring post-treatment, especially for those with minimal Cornell voltage reduction. These findings suggest that hypertension-induced irreversible myocardial damage, or the inability to regress LVH, may be a key contributor to elevated cardiovascular risk, particularly HF. Notably, intensive systolic BP reduction’s impact in SPRINT largely stemmed from HF reduction [15] —with irreversible hypertrophied myocardium often entailing permanent fibrosis and consequent impairment of LV function, leading to HF [7]. Thus, in persistent LVH, the probability for a larger proportion of irreversible myocardial injury is higher, and the degree of Cornell voltage regression will be smaller, which leads to a worse prognosis. On the other hand, LVH regression may improve coronary flow and reduce the risk of cardiac arrhythmia such as AF [33, 34], further contributing to a reduced risk of HF. Nevertheless, it is essential to acknowledge that the benefits of antihypertensive therapy in terms of cardiovascular events may necessitate a longer follow-up duration, and LVH regression could exert multifactorial effects on the cardiovascular system. Hence, further studies are imperative to evaluate the long-term effects of LVH regression and its pathophysiologic consequences on various cardiovascular outcomes.

### Clinical relevance

Our findings illuminate the complex interplay between hypertension and LVH. Emphasizing the prognostic value of LV geometric changes, especially Cornell voltage, this study advocates for a refined hypertension management strategy that accounts for LVH risk. Future long-term studies are essential to fully understand the enduring impacts of intensive BP control on the LVH landscape. Setting optimal BP goals may be key in curbing the incidence of new-onset LVH and reducing the overall burden of hypertrophy in hypertensive populations. Furthermore, our data indicate that LVH status changes, detectable via electrocardiography, are not only reflective of treatment efficacy but may also signal irreversible cardiac damage. Consequently, ECG monitoring of Cornell voltage and LVH could guide the management of hypertension, offering critical insights for optimizing care, particularly for those with persistent LVH who may benefit from more aggressive intervention strategies.

### Study limitations

Several limitations should be acknowledged as potential constraints in our study. Firstly, the representativeness of the study population warrants scrutiny. The demographic characteristics of our study cohort, marked by advanced age, absence of diabetes, and a notable burden of comorbidities, potentially curtail the generalizability of our results to more diverse demographic cohorts. Future investigations should contemplate the inclusion of participants with varied profiles to enhance the breadth of applicability. Secondly, we acknowledge that our study did not capture specific data on the duration of hypertension for either pre-existing or new-onset LVH patients. This information was not available in the dataset, limiting our ability to assess the impact of long-term hypertension on the development and progression of LVH. This gap reflects the retrospective nature of our study and the initial focus on other cardiovascular metrics, which precluded a comprehensive hypertension duration analysis. Thirdly, due to incomplete medication records in our dataset, a detailed analysis of pharmacological impacts on LVH was not feasible. This limitation underscores the need for future studies to incorporate comprehensive medication tracking to elucidate the effects of specific treatments on LVH progression and regression. Fourthly, the frequency of ECG recordings emerges as a noteworthy consideration. The lack of detailed interval data between the baseline and follow-up ECG assessments. Due to the retrospective design and the nature of the available data, we were unable to precisely adjust for the time intervals between ECGs as a factor in our analysis. This could influence our understanding of the dynamics of LVH progression or regression over time. Future prospective studies should consider including such temporal data to enhance the accuracy of similar analyses. Fifthly, the absence of cardiac imaging data underscores a critical limitation. The lack of information on cardiac imaging parameters within the SPRINT participants curtails a comprehensive understanding of structural changes, hindering additional insights into the intricate relationship between BP management and LVH. Lastly, the duration of follow-up surfaces as a critical consideration. The relatively abbreviated follow-up duration in our study may inadequately capture enduring effects and trends. Subsequent prospective studies, marked by extended observation periods, are imperative to comprehensively evaluate the enduring impact of intensive BP control on new-onset LVH and its associated outcomes.

## Conclusion

Our research demonstrates that intensive BP management can significantly reduce the onset of LVH among hypertensive patients, compared to standard treatment protocols. The study further highlights that stringent BP control can also promote regression in those with established LVH. Importantly, both new-onset and pre-existing LVH are associated with an elevated risk of adverse cardiovascular and safety events, as evidenced by the SPRINT data. Interestingly, the observed reduced correlation between LVH regression and cardiovascular risk suggests beneficial effects of intensive BP control. For patients at high risk, achieving BP levels below 120/80 mmHg is crucial for diminishing cardiovascular risk, highlighting the importance of optimal BP management in mitigating residual risk.

### Electronic supplementary material

Below is the link to the electronic supplementary material.

Supplementary Material 1

Supplementary Material 2

## Data Availability

All data and materials have been made publicly available at the National Heart, Lung, and Blood Institute and can be accessed at https://biolincc.nhlbi.nih.gov/studies/sprint/. Data are however available from the authors upon reasonable request and with permission from BioLINCC.
