# Supplementary material for: Electrocardiographic tracking of left ventricular hypertrophy in hypertension: incidence and prognostic outcomes from the SPRINT trial
Source: Clin Hypertens. 2024 Jul 1;30:17. doi: 10.1186/s40885-024-00275-8 (PMC11215828; doi:10.1186/s40885-024-00275-8)
Supplement: Supplementary file 1 — Supplementary Material 1 [file 40885_2024_275_MOESM1_ESM.doc]

**Supplemental Table S1.** Baseline characteristics

| Characteristics* | Free of LVH  (n=7100) | New-onset LVH  (n=319) | Pre-existing LVH  (n=597) | p | |
| --- | --- | --- | --- | --- | --- |
|  |  |  |  | New vs. No | Pre vs. No |
| Age, y [IQR] | 67.00 [61.00, 75.00] | 71.00 [62.00, 78.00] | 68.00 [60.00, 75.00] | <0.001 | 1 |
| Age ≥75 y, n (%) | 1924 (27.1) | 125 (39.2) | 168 (28.1) | <0.001 | 1 |
| Female, n (%) | 2302 (32.4) | 175 (54.9) | 353 (59.1) | <0.001 | <0.001 |
| Black, n (%) | 2044 (28.8) | 148 (46.4) | 309 (51.8) | <0.001 | <0.001 |
| Current Smoking, n (%) | 959 (13.5) | 40 (12.6) | 82 (13.8) | 1 | 1 |
| Alcohol Abuse, n (%) | 284 (4.0) | 8 (2.5) | 14 (2.3) | 0.700 | 0.170 |
| Body mass index, kg/m2 [IQR] | 29.04 [25.96, 32.88] | 28.57 [25.74, 33.40] | 29.06 [25.53, 32.95] | 1 | 1 |
| Systolic blood pressure, mm Hg [IQR] | 138.00 [129.00, 148.00] | 138.00 [129.00, 150.00] | 144.00 [134.00, 158.00] | 1 | <0.001 |
| Diastolic blood pressure, mm Hg [IQR] | 78.00 [70.00, 86.00] | 76.00 [68.00, 84.00] | 80.00 [71.00, 88.00] | 0.062 | <0.001 |
| Blood pressure medications, n | 2.00 [1.00, 2.00] | 2.00 [1.00, 3.00] | 2.00 [1.00, 3.00] | <0.001 | <0.001 |
| Serum creatinine, mg/dL [IQR] | 1.01 [0.86, 1.20] | 0.99 [0.82, 1.22] | 0.99 [0.83, 1.23] | 1.00 | 0.280 |
| Estimated GFR, mL/min/ 1.73m2 [IQR] | 71.53 [58.76, 84.74] | 70.00 [54.62, 84.59] | 70.29 [56.82, 84.03] | 0.150 | 0.150 |
| Urine albumin/creatinine, mg/g [IQR] | 9.18 [5.56, 20.00] | 11.04 [6.07, 24.88] | 13.01 [6.94, 32.66] | 0.032 | <0.001 |
| Chronic kidney disease†, n (%) | 1918 (27.0) | 117 (36.7) | 181 (30.3) | <0.001 | 0.2712 |
| Total cholesterol, mg/dL [IQR] | 186.00 [161.00, 214.00] | 191.00 [162.00, 216.00] | 191.00 [166.00, 222.00] | 1 | 0.004 |
| High-density lipoprotein cholesterol, mg/dL [IQR] | 50.00 [43.00, 60.00] | 52.00 [44.00, 64.00] | 53.00 [44.00, 64.00] | 0.020 | <0.001 |
| Triglycerides, mg/dL [IQR] | 108.00 [78.00, 152.00] | 100.00 [74.50, 140.50] | 99.00 [74.00, 138.00] | 0.050 | 0.001 |
| Fasting plasma glucose, mg/dL [IQR] | 98.00 [91.00, 105.00] | 96.00 [88.00, 103.00] | 96.00 [90.00, 104.00] | 0.003 | 0.015 |
| Clinical/subclinical cardiovascular disease, n (%) | 1345 (18.9) | 78 (24.5) | 162 (27.1) | 0.053 | <0.001 |
| Heart Failure, n (%) | 204 (2.9) | 28 (8.8) | 33 (5.5) | <0.001 | 0.002 |
| Atrial Fibrillation, n (%) | 528 (7.4) | 45 (14.2) | 54 (9.1) | <0.001 | 0.5327 |
| Statin Use, n (%) | 3148 (44.6) | 130 (41.0) | 228 (38.5) | 0.696 | 0.015 |
| Aspirin Use, n (%) | 3656 (51.6) | 157 (49.5) | 289 (48.5) | 1 | 0.480 |
| Cornell voltage,μV [IQR] | 1407.00 [1051.00, 1765.25] | 1975.00 [1610.50, 2174.50] | 2858.00 [2452.00, 3178.00] | <0.001 | <0.001 |

*Categorical variables are represented as counts with proportions and continuous variables are represented as medians with interquartile ranges.

†Defined as baseline estimated glomerular filtration rate< 60 ml/min/1.73m2

**Supplemental Table S2. Effect of intensive blood pressure lowering on the risk of developing new-onset** LVH and LVH regression in SPRINT participants using different ECG criteria for LVH

| Outcome | Intensive SBP lowering | Standard SBP lowering | Standard versus Intensive SBP lowering | |
| --- | --- | --- | --- | --- |
|  | IR (95% CI) | IR (95% CI) | adjusted IRR (95% CI) | p |
| **New-onset LVH** |  |  |  |  |
| Cornell voltage | 8.27(6.85,9.88) | 14.79(12.85,16.91) | 1.57(1.24,2.01) | 2.53×10^-4^ |
| Cornell voltage product | 14.15(12.25,16.24) | 23.3(20.83,25.96) | 1.45(1.21,1.74) | 8.06×10^-5^ |
| Minnesota Code | 3.91(2.97,5.04) | 6.42(5.18,7.84) | 2.01(1.01,2.02) | 4.73×10^-2^ |
|  |  |  |  |  |
| **LVH regression** |  |  |  |  |
| Cornell voltage | 14.89(12.96,17.01) | 11.89(10.16,13.80) | 0.71(0.57,0.88) | 1.56×10^-3^ |
| Cornell voltage product | 22.32 (19.92,24.92) | 19.99(17.71,22.46) | 1.00(0.71,1.00) | 5.55×10^-2^ |
| Minnesota Code | 6.33(5.1,7.73) | 4.42(3.41,5.62) | 0.85(0.43,0.85) | 3.75×10^-3^ |

Abbreviations: CI, confidence interval; ECG, electrocardiogram; LVH, left ventricular hypertrophy; IR, incidence rate; IRR, incidence rate ratio; SBP, systolic blood pressure.

**Supplemental Table S3. Prognostic Implications of New-onset and Pre-existing LVH on cardiovascular and safety outcomes in patients using different ECG criteria for LVH**

| Endpoints | Cornell voltage product LVH criteria | | Minnesota Code LVH criteria | |
| --- | --- | --- | --- | --- |
|  | HR(95% CI) | P value | HR(95% CI) | P value |
| **ACE** |  |  |  |  |
| preLVH | 1.45 [1.02, 2.04] | 0.0359 | 0.90 [0.51, 1.61] | 0.7333 |
| newLVH | 1.29 [0.91, 1.84] | 0.1583 | 2.36 [1.51, 3.69] | 0.0002 |
| **Stroke** |  |  |  |  |
| preLVH | 1.33 [0.65, 2.72] | 0.4398 | 1.52 [0.58, 3.97] | 0.3925 |
| newLVH | 0.72 [0.28, 1.82] | 0.4852 | 1.40 [0.43, 4.54] | 0.576 |
| **MI** |  |  |  |  |
| preLVH | 1.62 [0.93, 2.85] | 0.0898 | 1.12 [0.48, 2.62] | 0.8019 |
| newLVH | 1.49 [0.87, 2.56] | 0.1464 | 2.05 [0.94, 4.45] | 0.0694 |
| **NONMIACS** |  |  |  |  |
| preLVH | 1.44 [0.60, 3.44] | 0.418 | 0.72 [0.16, 3.19] | 0.6662 |
| newLVH | 2.19 [1.03, 4.64] | 0.0407 | 1.30 [0.31, 5.42] | 0.7224 |
| **HF** |  |  |  |  |
| preLVH | 1.50 [0.82, 2.75] | 0.1879 | 0.94 [0.33, 2.68] | 0.9018 |
| newLVH | 1.43 [0.79, 2.60] | 0.2421 | 3.83 [2.06, 7.12] | <0.0001 |
| **CV Death** |  |  |  |  |
| preLVH | 3.61 [1.17, 11.10] | 0.0254 | 0.00 [0.00, Inf] | 0.9956 |
| newLVH | 1.95 [0.63, 6.04] | 0.2497 | 1.13 [0.15, 8.56] | 0.9085 |
| **Death** |  |  |  |  |
| preLVH | 1.62 [0.87, 3.04] | 0.131 | 0.82 [0.28, 2.36] | 0.7135 |
| newLVH | 0.87 [0.41, 1.82] | 0.7082 | 1.83 [0.73, 4.57] | 0.1986 |
| **ACE or Death** |  |  |  |  |
| preLVH | 1.34 [0.97, 1.86] | 0.0753 | 0.98 [0.58, 1.66] | 0.9382 |
| newLVH | 1.13 [0.80, 1.59] | 0.4938 | 2.32 [1.52, 3.56] | 0.0001 |
| **Safety endpoint** |  |  |  |  |
| preLVH | 1.20 [1.05, 1.38] | 0.0097 | 1.20 [0.96, 1.50] | 0.105 |
| newLVH | 1.28 [1.11, 1.47] | 0.0008 | 1.31 [1.02, 1.68] | 0.0314 |

newLVH refers to new-onset LVH; preLVH refers to pre-existing LVH. Definitions of each LVH group can be found in Method section.

Abbreviations: HR, hazard ratio; CI, confidence interval; LVH, left ventricular hypertrophy; ACE, adverse cardiovascular events; HF, heart failure; MI, myocardial infarction; NOMIACS, non-MI acute coronary syndrome; CV, Cardiovascular.

**Supplemental Table S4. Effects of intensive blood pressure lowering on cardiovascular and safety outcomes stratified by LVH status using different ECG criteria for LVH**

| Endpoints | Cornell voltage product LVH criteria | | Minnesota Code LVH criteria | |
| --- | --- | --- | --- | --- |
|  | HR(95% CI) | P value | HR(95% CI) | P value |
| **ACE** |  |  |  |  |
| recovered LVH | 1.24 [0.83, 1.87] | 0.2971 | 0.60 [0.26, 1.37] | 0.2238 |
| persistent LVH | 1.49 [1.01, 2.21] | 0.0459 | 1.30 [0.60, 2.83] | 0.5019 |
| **Stroke** |  |  |  |  |
| recovered LVH | 1.20 [0.51, 2.83] | 0.6785 | 0.97 [0.23, 4.07] | 0.9661 |
| persistent LVH | 1.64 [0.73, 3.68] | 0.2338 | 2.36 [0.70, 7.99] | 0.1681 |
| **MI** |  |  |  |  |
| recovered LVH | 1.28 [0.64, 2.54] | 0.4817 | 0.84 [0.26, 2.69] | 0.7689 |
| persistent LVH | 1.71 [0.90, 3.24] | 0.1023 | 1.47 [0.45, 4.83] | 0.5227 |
| **NONMIACS** |  |  |  |  |
| recovered LVH | 0.75 [0.24, 2.38] | 0.6312 | 0.00 [0.00, Inf] | 0.9938 |
| persistent LVH | 1.65 [0.63, 4.32] | 0.3099 | 2.21 [0.49, 9.93] | 0.299 |
| **HF** |  |  |  |  |
| recovered LVH | 1.45 [0.73, 2.87] | 0.2915 | 0.63 [0.15, 2.70] | 0.5332 |
| persistent LVH | 1.28 [0.64, 2.55] | 0.4825 | 1.06 [0.25, 4.53] | 0.9329 |
| **CV Death** |  |  |  |  |
| recovered LVH | 2.60 [0.69, 9.75] | 0.157 | 0.00 [0.00, Inf] | 0.9964 |
| persistent LVH | 3.53 [1.05, 11.84] | 0.0412 | 0.00 [0.00, Inf] | 0.9975 |
| **Death** |  |  |  |  |
| recovered LVH | 1.38 [0.64, 3.01] | 0.4124 | 0.81 [0.24, 2.74] | 0.7323 |
| persistent LVH | 1.95 [0.96, 3.94] | 0.0643 | 0.74 [0.10, 5.45] | 0.7665 |
| **ACE or Death** |  |  |  |  |
| recovered LVH | 1.23 [0.84, 1.81] | 0.293 | 0.68 [0.33, 1.40] | 0.2993 |
| persistent LVH | 1.39 [0.96, 2.03] | 0.0842 | 1.39 [0.67, 2.87] | 0.3775 |
| **Safety endpoint** |  |  |  |  |
| recovered LVH | 1.09 [0.92, 1.28] | 0.3314 | 1.25 [0.96, 1.62] | 0.104 |
| persistent LVH | 1.22 [1.03, 1.43] | 0.0204 | 1.07 [0.74, 1.56] | 0.7059 |

recovered LVH refers to LVH regression. Definitions of each LVH group can be found in Method section.

Abbreviations: HR, hazard ratio; CI, confidence interval; LVH, left ventricular hypertrophy; ACE, adverse cardiovascular events; HF, heart failure; MI, myocardial infarction; NOMIACS, non-MI acute coronary syndrome; CV, Cardiovascular.
