# Supplementary material for: Electrocardiographic tracking of left ventricular hypertrophy in hypertension: incidence and prognostic outcomes from the SPRINT trial
Source: Clin Hypertens. 2024 Jul 1;30:17. doi: 10.1186/s40885-024-00275-8 (PMC11215828; doi:10.1186/s40885-024-00275-8)
Supplement: Supplementary file 2 — Supplementary Material 2 [file 40885_2024_275_MOESM2_ESM.doc]

/

**Fig S1.** Cornell voltage in the two treatment groups in patients with or without LVH at baseline. I bars represent 95% confidence intervals. CV, Cornell voltage; LVH, left ventricular hypertrophy. preLVH refers to pre-existing LVH.
